# G-protein inwardly rectifying potassium channel 1 (GIRK 1) gene expression correlates with tumor progression in non-small cell lung cancer

**DOI:** 10.1186/1471-2407-4-79

**Published:** 2004-11-13

**Authors:** Iwao Takanami, Yoshimasa Inoue, Masatoshi Gika

**Affiliations:** 1 First Department of Surgery, Teikyo University School of Medicine, Tokyo, Japan

## Abstract

**Background:**

G-protein inwardly rectifying potassium channel 1 (GIRK1) is thought to play a role in cell proliferation in cancer, and GIRK1 gene expression level may define a more aggressive phenotype. We detected GIRK1 expression in tissue specimens from patients with non-small cell lung cancers (NSCLCs) and assessed their clinical characteristics.

**Methods:**

Using reverse transcription-polymerase chain reaction (RT-PCR) analyses, we quantified the expression of GIRK1 in 72 patients with NSCLCs to investigate the relationship between GIRK1 expression and clinicopathologic factors and prognosis.

**Results:**

In 72 NSCLC patients, 50 (69%) samples were evaluated as having high GIRK1 gene expression, and 22 (31%) were evaluated as having low GIRK1 gene expression. GIRK1 gene expression was significantly associated with lymph node metastasis, stage (p = 0.0194 for lymph node metastasis; p = 0.0207 for stage). The overall and stage I survival rates for patients with high GIRK1 gene expressed tumors was significantly worse than for those individuals whose tumors had low GIRK1 expression (p = 0.0004 for the overall group; p = 0.0376 for stage I).

**Conclusions:**

These data indicate that GIRK1 may contribute to tumor progression and GIRK1 gene expression can serve as a useful prognostic marker in the overall and stage I NSCLCs.

## Background

Lung cancer is one of the leading causes of cancer death in North America [1]. Lung cancer is divided into two morphological types: small cell lung cancer (SCLC) and non-small cell lung cancer (NSCLC). The results of surgery still remain unsatisfactory; even in stage I NSCLC (no lymph node metastasis and no distant metastasis) about 30% of patients die due to disease recurrence within 5 years after curative resection [2]. Despite major advances in cancer treatment in the past decades, the prognosis of patients with NSCLC has improved only minimally [1]. A new knowledge of the molecular pathogenesis of cancer has emerged from investigative advances in the field of molecular biology [3]. Increased knowledge of the biologic role of genetic changes has provoked an intriguing search for clinical applications of these alterations [4], and has enabled the more aggressive tumors from the less aggressive ones to be distinguished [5].

G-protein inwardly rectifying potassium channels (GIRK) are found in both the heart and the brain, where they are associated with a slowing of the heart rate and suppression of neuronal response [6]. The channel found in the sinoatrial node and on atrial myocytes is formed from the homologous channel subunits GIRK1 and GIRK4 [7]. Although the function of GIRK1 still remains unclear except in cell proliferation in cancer, GIRK1 gene expression has been found to correlate with lymph node metastasis in breast carcinomas [8], but the correlation with GIRK1 gene expression and prognosis has never been analyzed in NSCLC. To our knowledge, this is the first report to analyze the prognostic influence of GIRK1 gene expression in NSCLC and the possible associations between this parameter and other clinical factors. In this study, we used RT-PCR for detecting GIRK1 in tumor tissues. We compared with GIRK1 gene expression with autocrine motility factor-receptor (AMF-R) gene expression, known as a marker of lymph node metastasis and tumor progression, and we investigated how GIRK1 gene expression is related to tumor progression and prognosis in a series of 72 cases of curatively resected NSCLC.

## Methods

### Tissue specimens

Tumor tissue was collected from 72 patients with NSCLC who underwent curative surgery between 1993 and 1995 at Department of Surgery, Teikyo University School of Medicine. Patients who died within one month after surgery and patients with a past history of another cancer were excluded from the study. Of the 72 patients included, 52 were men and 20 were women and their ages ranged from 34 to 80 years (mean of 66 years). With regard to histological type, 41 were adenocarcinomas, 28 were squamous cell carcinomas and 3 were large cell lung carcinomas. The lesions of these 72 patients were staged on both operative and pathologic findings according to the UICC TNM classification (1997) [9]. There were 24 patients with stage IA, 11 patients with stage IB, 1 patients with stage IIA, 13 patients with stage IIB, and 23 patients with stage IIIA. These patients were performed curative operation with lymph node dissection. The mean follow-up time was 52.0 months (range, 2.7–120.0 months). Freshly removed pulmonary cancer tissues for RNA extraction were immediately frozen in liquid nitrogen and stored at -80°C until further use. And in five of the cases, adjacent normal pulmonary material from the same patient was also used in this study. Tissue samples to be used for hematoxylin-and-eiosin were fixed in formalin and paraffin embedded.

### Reverse transcription-polymerase chain reaction (RT-PCR) analysis

Total RNA was purified from fresh soft tissues by the acid guanidinium-thiocyanate procedure [10]. The human pulmonary adenocarcinoma cell line PC-14 (Riken Gene Bank Co., Ltd. Tokyo, Japan) was used as a positive control. All the RNA (5 μg) was used for cDNA synthesis, and the first-standard cDNA solution was then used for the PCR, with primers designed to amplify a 230 bp sequence (sense primer sequence: 5'-GGGATTTGGACATGGCTAAGTC-3' ; antisense primer sequence: 5'-GGCCTGTTTTCATTCTCTTAACTGATAC-3'). The reaction mixture was overlaid with 20 μl of mineral oil. PCR was performed for forty cycles (10 s at 95°C; 60 s at 60°C, and 120 s at 72°C) as previously described [8]. S14 cDNA amplification using the same temperature profile for 30 cycles served as the internal control [11]: the sense and antisense primers for S14 cDNA amplification were 5'-GGCAGACCGAGATGAATCCTC-3' and 5'-CAGGTCCAGGGGTCTTGGTCC-3'. The amplified DNA samples were electrophoresed on 1% agarose gels, and photographed with a Polaroid camera. Densitometric analysis of the photographic negatives was used for band quantification.

### Specimen classification based on RT-PCR results

The densitometric value obtained for the GIRK1 band of a given tumor tissue sample was divided by the corresponding S14 value, and was referred to as the GIRK1 gene expression rate. The level of the GIRK1 mRNA expression in PC-14 cell line is elevated. The expression ratio of the tumor was then divided by that of the human pulmonary adenocarcinoma cell line PC-14 to obtain the GIRK1 conservation rate. When the conservation rate of a given specimen was ≧ 0.8, it was considered to indicate high expression of the GIRK1 gene, and if the rate was < 0.8, it was defined as low expression.

### Comparison with GIRK1 gene expression and AMF-R gene expression

To ascertain whether links between GIRK1 gene expression and another gene expression, known as a marker of lymph node metastasis and tumor progression in NSCLC, we examined the relationships between GIRKI gene expression and AMF-R gene expression in 46 cases [12]. The studies of AMF-R gene expression were performed as described previously [12]. The expression ratio of the tumor was divided by that of the cell line PC-14, and the conservation rate of a given specimen was larger than the mean ratio, it was considered to indicate high expression of the AMF-R gene, and if the rate was lower than the mean ratio, it was defined as low expression of the AMF-R gene.

### Statistical analysis

All data regarding the clinical and histopathological variables were stored in a Macintosh computer. The Stat View program (Aracus Concepts, Berkeley, Ca, USA) was used for all statistical analyses. The relationship between the incidence of GIRK1 expression and clinico-pathologic factors, and AMF-R gene expression was examined by the chi-squared test with Fisher correlation. Survival curves were calculated using the Kaplan-Meier method and analyzed by the log-rank test. Statistical significance was identified as p < 0.05.

## Results

### Detection of GIRK1 using RT-PCR in NSCLC tissues

**Figure 1:**
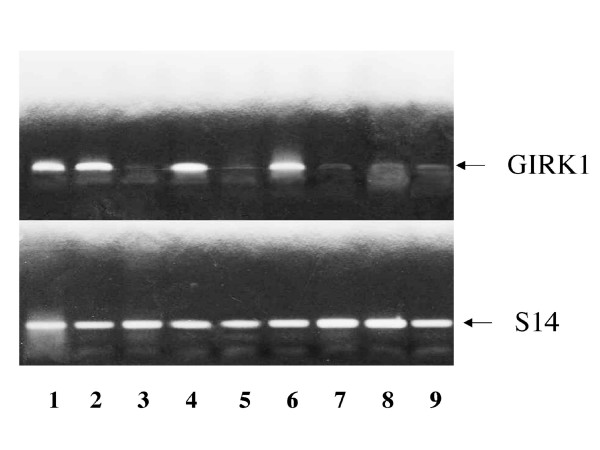
Agarose gel electrophoresis of RT-PCR amplified 230 bp GIRK1 cDNA, and 143 bp S14 DNA as internal PCR control. Lane 1, size marker; Lane 2, human pulmonary adenocarcinoma cell line PC-14 (positive control); Lane 3, pulmonary adenocarcinoma with high GIRK1 expression. Lane 4, pulmonary adenocarcinoma with low GIRK1 expression. Lane 5, squamous cell carcinoma with high GIRK1 expression. Lane 6, squamous carcinoma with low GIRK1 expression. Lane 7, large cell carcinoma with high GIRK1 expression. Lane 8, large cell carcinoma with low GIRK1 expression. Lane 9, and Lane 10, normal pulmonary tissue with low GIRK1 expression.

To determine the number of PCR cycles appropriate for quantification, from 20 and 50 cycles of PCR were performed, in 5-cycle increments. The expression ratios of GIRK1 to S14 were reasonably constant 35 to 45 cycles (data not shown). Therefore, in the subsequent experiments the values obtained at 40 cycles were defined as the expression of the target genes. Using forty RT-PCR cycles, we found that the ratio of GIRK1/cell line PC-14 expression ranged from 0 to 2.2 (means, 0.8) in tumor specimens (Fig. 1). Of 72 NSCLCs studied, 50 (69%) were classified as GIRK1 high gene expression, and 22(31%) were classified as having GIRK1 low gene expression. Five adjacent normal pulmonary materials ranged from 0 to 0.1 (means = 0.1), and all of them were classified as GIRK1 low gene expression.

### Relationship between GIRK1 gene expression and clinicopathological factors

The relationships between GIRK1 gene expression and various clinicopathological factors are shown in Table 1. There were no statistically significant relationships between gene expression and age, gender, T factor and histology. In contrast, GIRK1 gene expression was associated with N factor and stage (p = 0.0194 for lymph node metastasis, p = 0.0207 for stage).

### The relationships between GIRK1 gene expression and AMF-R gene expression

Of 46 NSCLCs studied, 33 (72%) were classified as AMF-R high gene expression, and 13(28%) were classified as having AMF-R low gene expression. In most GIRK1 high gene expression cases, AMF-R gene was also expressed. As shown Table 2, the results of GIRK1 gene expression agreed significantly with AMF-R gene and 72% of cases had no discrepancy (p = 0.0303).

### Association of tumor GIRK1 gene expression and survival

**Figure 2:**
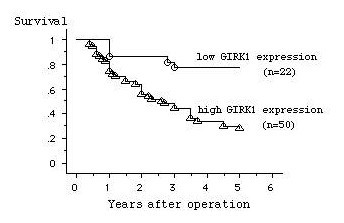
Overall survival of 72 lung cancer patients according to GIRK1 amplification. Survival curves were calculated by the Kaplan-Meier method, and statistical evaluation was determined by the log-rank test (p = 0.0004).

**Figure 3:**
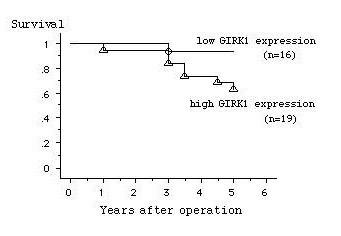
Survival curves of the patients with stage I NSCLC on the basis of GIRK1 amplification. A significant difference was seen between the 2 groups (p = 0.0376).

**Figure 4:**
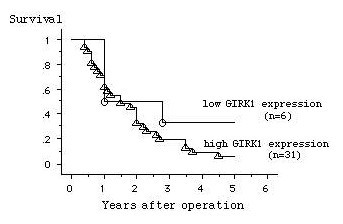
Survival curves of the patients with stage II / III NSCLC on the basis of GIRK1 amplification. A significant difference was not seen between the 2 groups.

The survival was compared between the high GIRK1 gene expression group and the low GIRK1 gene expression group in overall, stage I and stage II/III. Figures 2 and 3 show the significance in survival between the high GIRK1 gene expression group and the low GIRK1 gene expression group in overall and stage I groups (P = 0.0004 for the overall group; P = 0.0376 for the stage I group). Although the 5-year survival in low GIRK1 gene expression group is better than that in the high GIRK1 gene expression group in the stage II/III, there is not a difference in survival between the 2 groups (Figure 4).

## Discussion

In spite of significant advances in surgery and the use of new, more effective chemotherapeutic regimens, the overall 5-year survival of patients with NSCLC is 17% [13]. Identification of new prognostic factors might be of value in directing therapy and intensifying follow-up for a select group of patients. Lymph node metastasis and stage are the most powerful prognostic markers for NSCLC. Identifying new genes that are associated with tumor growth, metastasis, and prognosis is very important in advancing the understanding of cancer biology.

Human carcinomas exhibit hyperpolarized membrane potential as compared with surrounding normal tissue [14,15]. GIRK1 acts to conduct potassium ions into the cell rather than out of the cell, and play a role in maintaining membrane potential. Though GIRK1 act to hyperpolarizing the cell membrane, the function of GIRK1 still remains completely unclear in cancer. Cell proliferation and the density of intracellular potassium are controlled at specific stages of the cell cycle [16], and cell membrane potential indeed changes during the cell cycle [14]. And GIRK1 is reported to play a role in cell proliferation [3]. Receptors known to activate GIRK1 belong to the family of G-protein coupled receptors, and the G-protein-coupled receptors are reported to be able to induce cell proliferation and activate a pathway leading to angiogenesis in tumor [17]. GIRK1 gene overexpression is reported to follow a general trend of increasing expression if lymph node metastasis is involved in breast carcinomas [8]. Though the mechanical role of GIRK1 in lymph node metastasis in cancer is not clear, angiogenesis is reported to be correlated with lymph node metastasis, tumor progression and poor prognosis in most of solid tumors [18,19]. Therefore, GIRK1 is thought to be able to act not only in cell proliferation but also as an angiogenesis activator as well as G-protein-coupled receptors. S-kinase-associated protein 2 (Skp2) plays a critical role in regulating cell cycle progression and human factor-8-related antigen (F8RA) is assessed to show angiogenesis by microvessel density. So we determined whether or not expression of GIRK1 mRNA correlated with immunohistochemical assays of Skp2 and F8RA. Patients with high expression of GIRK1 mRNA were tendency to show high MVD and positive Skp2 expression without significance (data not shown). GIRK1 could be a candidate for a pharmaceutical target, depending upon further functional studies.

In this study, we used human pulmonary adenocarcinoma cell line PC-14 as a positive control for GIRK1 gene expression. The patients were classified into two groups according to the cutoff point of mean ration of GIRK1/cell line PC-14 expression in tumor specimens. The mean number has been widely used as the cutoff point to divide the patients into two groups [20,21]. GIRK1 was expressed at higher levels in cancer tissue than in adjacent normal lung tissue. It was shown that a high GIRK1 gene expression was detected in 69% of the tumor samples in our patient population with NSCLC. Furthermore, GIRK1 gene expression was also associated with nodal status, and tumor stage. These results, in correlation with nodal status, were similar to a previous report on breast carcinomas [8]. We examined the relationships between GIRKI gene expression and AMF-R gene expression, known as a marker of lymph node metastasis and tumor progression in NSCLC [12], and the results of GIRK1 gene expression agreed significantly with AMF-R. Statistical associations between GIRK1 expression and clinicopathological variables (age, T-factor, histology, and AMF-R) were examined by regression analysis. This analysis also showed that GIRK1 was correlated with AMF-R (data not shown). It was observed that patients with high GIRK1 expression NSCLC showed an unfavorable prognosis compared with those whose tumors had low GIRK1 expression in overall. Many patients in stage II/III disease had high GIRK1 expression than low GIRK1 expression. Therefore the poorer survival in overall was possible to be due to stage. So we compared patients in each stage, and we found a positive correlation between GIRK1 expression and surgical outcome in stage I cancer but not a positive correlation in stage II/III disease. Our results suggest that a high GIRK1 gene expression was strongly associated with an increased recurrence in stage I cancer and that patients with high GIRK1 gene expression may be prone to metastasis, or may already have occult micrometastasis to the lymph node in stage I cancer. On the other hand, GIRK1 expression does not seem to be a prognostic predictor for stage II/III disease individuals. GIRK1 gene expression level may play one of a key role in the biology of lung cancer and define a more aggressive tumor phenotype. Further studies are needed on GIRK1 to evaluate the mechanism of GIRK1 and more studies with a larger group of patients will be necessary to substantiate these data. A real quantative PCR amplication is now the standard approach, and more sensitive and accurate than RT-PCR. We would use the real-time quantative PCR amplication instead of RT-PCR in the next study for estimating the gene expression.

In conclusion, the present study suggests GIRK1 may be contribute to tumor progression and could be a useful prognostic marker in patients with overall and stage I NSCLC. Thus the current findings provide evidence to support a potential utility of this gene in developing a diagnostic test for NSCLC patients.

## Competing interests

The author(s) declare that they have no competing interest.

## Authors' contributions

IT: Data analysis and writing of manuscript. YI, MG: Critical appraisal of manuscript. All authors read and approved the final manuscript.

## Pre-publication history

The pre-publication history for this paper can be accessed here:



## Figures and Tables

**Table 1:** Correlation between GIRK1 gene expression and various clinicopathologic factors in patients with lung cancer

Variable	GIRK1		p value
		
	Low expression (n = 22) (31%)	High expression (n = 50) (69%)	
Age at surgery (yrs)			
≤60	8 (11 %)	19 (26 %)	
>60	14 (20 %)	31 (43 %)	0.90
Sex			
Male	17 (24 %)	35 (48 %)	
Female	5 (7 %)	15 (21 %)	0.53
Tumor status			
T1	10 (14 %)	11 (15 %)	
T2	10 (14 %)	25 (35 %)	0.07
T3	2 (3 %)	14 (19 %)	
Nodal status			
N0	17 (24 %)	26 (36 %)	
N1	3 (4 %)	3 (4 %)	0.02
N2	2 (3 %)	21 (29 %)	
Stage			
IA/IB	16 (22 %)	19 (26 %)	
IIA/IIB	4 (6 %)	10 (14 %)	0.02
III A	2 (3 %)	21 (29 %)	
Histology			
Adenocarcinoma	15 (21 %)	26 (36 %)	
Squamous cell carcinoma	6 (8 %)	22 (30 %)	0.40
Large cell carcinoma	1 (2 %)	2 (3 %)	

**Table 2:** Relationship between GIRK1 gene expression results and autocrine motility factor receptor (AMF-R) gene expression results

	GIRK1 low expression	GIRK1 high expression	Total
AMFR low expression	7 (15 %)	6 (13 %)	13 (28 %)
AMFR high expression	7 (15 %)	26 (57 %)	33 (72 %)

P = 0.03
